# Hyperpolarized [U‑^2^H, 2-^13^C]Fructose Distinguishes Direct Hepatic Gluconeogenesis Through Fructose-1-Phosphate Production in Fed and Fasted States

**DOI:** 10.1021/acschembio.5c00980

**Published:** 2026-03-04

**Authors:** Celia Martínez de la Torre, Grace Figlioli, Mario C. Chang, Quinlan Cullen, Kayvan R. Keshari

**Affiliations:** † Department of Radiology, 5803 Memorial Sloan Kettering Cancer Center, New York, New York 10065, United States; ‡ Molecular Pharmacology Program, Memorial Sloan Kettering Cancer Center, New York, New York 10065, United States; § Weill Cornell Medical College, New York, New York 10065, United States

## Abstract

Hepatic fructose utilization depends on ketohexokinase mediated phosphorylation to generate fructose-1-phosphate and commit fructose carbons to additional metabolic steps. Since dysregulated fructose metabolism has been directly connected to the onset and progression of liver disease and cancer, there is considerable interest in identifying the contributions of fructose carbons in bioenergetic pathways. An essential technology for assessing fructose utilization has been the application of isotopically labeled fructose and magnetic resonance with the development of ^13^C hyperpolarized imaging with [2-^13^C]­fructose allowing for *in vivo* assessments. While hyperpolarized imaging of [2-^13^C]­fructose has achieved remarkable success in the detection of cancer metabolism, this approach has yet to be utilized to assess fed and fasted states in healthy livers. By challenging mice with a 6 h fast, we demonstrate that hyperpolarized [U-^2^H, 2-^13^C]­fructose *in vivo* spectroscopy can clearly distinguish direct hepatic gluconeogenesis. Comprehensively, this work aims to establish a foundational methodology for the assessment of hepatic metabolism *in vivo*.

## Graphical abstract

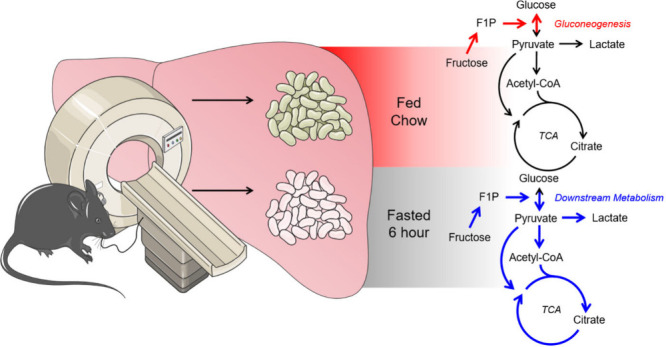

Over the last 70 years, efforts to assess metabolic utilization of isotopically labeled fructose have elucidated important insights into hepatic metabolism.^1^ The liver is a primary site of fructose metabolism, where hepatocytes import fructose predominantly through GLUT2 (SLC2A2) transporters. Fructose is then rapidly converted to fructose-1-phosphate (F1P) through ketohexokinase (KHK) activity^2,3^ and subsequently routed through aldolase B which converts F1P into dihydroxyacetone phosphate (DHAP) and glyceraldehyde-3-phosphate (GA3P).^4^ DHAP and GA3P can then be utilized for biosynthetic processes like gluconeogenesis and *de novo* lipogenesis or, alternatively, anaplerotic catalysis into the TCA cycle to sustain oxidative metabolism and cellular respiration.^5–8^

Given that dysregulated fructose metabolism has been heavily implicated in widely spread diseases like cancer and metabolic dysfunction-associated steatotic liver disease (MASLD),^9–13^ there has been substantial interest in understanding the contributions of fructose carbons in biosynthetic and oxidative pathways. An essential tool for quantifying these contributions is the use of ^13^C labeled fructose and analysis by nuclear magnetic resonance (NMR). Not only has ^13^C NMR analysis of fructose utilization effectively reported quantitative measures of both gluconeogenic and oxidative fractions,^4^ but it has also paved the way for the development of *in vivo* hyperpolarized [2-^13^C]­fructose imaging.

In 2009, Keshari et al. demonstrated the application of hyperpolarized [2-^13^C]­fructose to image the uptake and metabolism of fructose in a model of prostate cancer.^14^ Since then, further developments have significantly enhanced the capabilities of hyperpolarized [2-^13^C]­fructose imaging, primarily through the deuteration of fructose and the dissolution of hyperpolarized sample in D_2_O which have significantly increased the polarization and *T*_1_ of the fructose probe.^2,15^ Although this tracing approach has allowed for the detection of fructose metabolism in tumors, the effects of fed and fasted states on healthy livers have yet to be tested with hyperpolarized [2-^13^C]­fructose.

The liver is a complex organ capable of distinct metabolic and physiological responses triggered by feeding and fasting.^16,17^ In the case of glucose utilization, insulin and glucagon levels regulate metabolism during fed and fasted states.^16,18,19^ Feeding leads to an insulin response that stimulates glucose uptake and oxidation, while fasting triggers a glucagon response that stimulates gluconeogenesis in the liver. Unlike glucose, fructose uptake and metabolism are not governed by hormonal regulation^20^ and its exact metabolic fate during feeding and fasting is still not fully understood. One possibility is that fructose follows the same fate as glucose. A second possibility is that fructose follows the opposite fate and serves as a compensatory fuel to maintain processes alternating with the hormonally regulated effects of glucose metabolism. For example, when insulin triggers glucose uptake and oxidation, fructose can be utilized to replenish gluconeogenic precursors. When glucagon triggers gluconeogenesis, fructose can be routed to downstream metabolism to maintain the energetic demands of gluconeogenesis.

In the case of glucose, metabolic responses to both states have been studied extensively by probing the liver with ^13^C labeled substrates like glucose and pyruvate, and using a wide variety of analytical approaches including NMR and hyperpolarization.^21–25^ While these glycolytic studies are able to clearly distinguish increased hepatic gluconeogenesis in the fasted state and increased anaplerosis in the fed state,^21^ using either glucose or pyruvate as metabolic probes comes with limitations that hinder clinical translation and applicability. First, the application of a ^13^C labeled glucose probe is limited to infusion studies since its *T*_1_ is too short to be utilized as an effective hyperpolarization probe.^26^ Second, while hyperpolarized pyruvate has achieved unprecedented success as a noninvasive imaging probe in humans,^27,28^ it has limited physiological relevance as it requires bolus administration at mM concentrations which vastly supersede plasma concentrations in the μM range. Therefore, hyperpolarized [U-^2^H, 2-^13^C]­fructose may be a more favorable probe for distinguishing fed and fasted hepatic metabolism since it has a relatively long *T*_1_ of 60–100 s and can be administered at physiological mM concentrations.^2,14,15^

Here we investigate the capability of using hyperpolarized [U-^2^H, 2-^13^C]­fructose to noninvasively identify distinct hepatic metabolism in fed and fasted states using HP MRI. We demonstrate that hyperpolarized [U-^2^H, 2-^13^C]­fructose spectroscopy is sensitive to KHK activity and prandial states. More specifically, we show that in contrast to glucose metabolism, the fed liver utilizes fructose as a direct gluconeogenic source^29^ while the fasted liver oxidizes fructose. Overall, this study aims to establish a foundational methodology for the assessment of *in vivo* liver metabolism.

## Synthesis and Hyperpolarization of [U-^2^H, 2-^13^C]­Fructose

We utilized a modified version of the deuteration method developed by our laboratory for the synthesis of [U-^2^H, 2-^13^C]­fructose.^2^ In brief, and similar to other sugars, we utilized a Ru/C catalyst under H_2_ gas (1 atm) with D_2_O as the deuterium source.^30^ As previously reported, the site selectivity is possible by the protection of the hydroxyl group in the second carbon as acetals, preventing hydrolysis and forcing fructose to be in a 5-member ring (Figure 1A). Deuteration was performed in two to three 4 h cycles which achieved comparable levels of deuteration (≥90%) and faster total reaction times of 12–18 h (Figure 1A and Figures S1–S8). Figure 1B depicts the reaction scheme of KHK which generates [U-^2^H, 2-^13^C]­F1P from [U-^2^H, 2-^13^C]­fructose. Dissolution of hyperpolarized [U-^2^H, 2-^13^C]­fructose in D_2_O yielded equivalent *T*_1_ relaxation times across the α-furanose, β-furanose, and β-pyranose forms, indicating equilibrated exchange among the three isoforms (Figure 1C,D). Relative quantification of the percentage contribution of each isoform determined the presence of 70% pyranose and 30% furanose forms. Compared to previous attempts of hyperpolarizing protonated [2-^13^C]­fructose with H_2_O solvation, *T*_1_ relaxation measurements (65.4 ± 1.25 s) were increased by over 3.5×, which is consistent with other reports of hyperpolarized [U-^2^H, 2-^13^C]­fructose with D_2_O solvation.^2,15^ Compared to previous preparations of hyperpolarized [U-^2^H, 2-^13^C]­fructose, this preparation explored expedited probe production by assessing polarization without HPLC purification which substantially reduced costs and yield loss, although at the expense of some *T*_1_.

**Figure 1:**
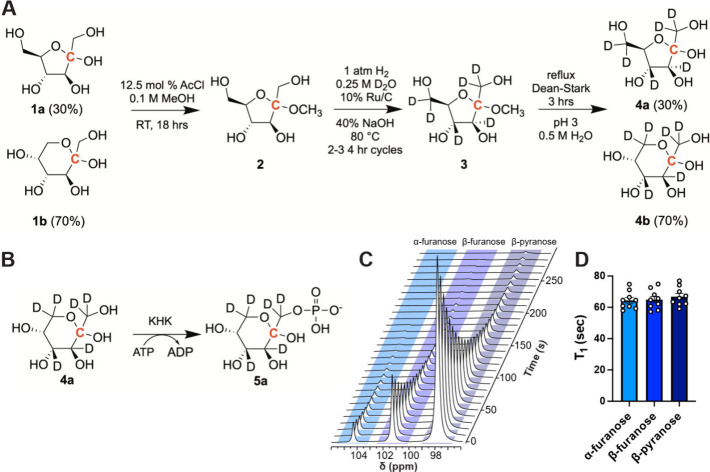
Synthesis and application of hyperpolarized [U-^2^H, 2-^13^C]­fructose as a metabolic probe. (A) Schematic representation of each step of the chemical synthesis of [U-^2^H, 2-^13^C]­fructose which involves protection, deuteration, and deprotection reactions. (B) Metabolic reaction scheme of ketohexokinase (KHK) activity highlights phosphorylation of the hydroxyl at the first carbon position of [U-^2^H, 2-^13^C]­fructose. (C) Representative pseudo 2D dynamic spectroscopy of hyperpolarized [U-^2^H, 2-^13^C]­fructose dissolved in D_2_O. (D) *T*_1_ relaxation measurements of the three predominant forms of [U-^2^H, 2-^13^C]­fructose – α-furanose, β-furanose, and β-pyranose.

## Increased F1P Accumulation in Fed Livers Detected with ^13^C Hyperpolarized MRS

The effects of fasting on fructose metabolism were tested by first allowing mice to have *ad libitum* access to food or, in another group, removing food access for 6 h (Figure 2A). A 6 h fast was chosen based on recent literature reporting that the use of human fasting times in mice (∼24 h) led to several confounding variables in the form of long-lasting metabolic adaptations like torpor, hypothalamic epigenetic changes, and impaired beta cell function as examples.^12,31–34^ These findings suggested that in mice, a 6 h fast better recapitulates the effects of a 24 h fast in humans. Therefore, when comparing the effects of fasting in metabolism, it is important to only make definitive conclusions when comparing fasts of equal lengths. After they fasted, mice were entered into the study and prepared for analysis by magnetic resonance imaging and spectroscopy. Standard *T*_2_ weighted ^1^H imaging sequences were utilized to acquire anatomical references to determine the localization of the liver (Figure 2B). After imaging, mice were injected with hyperpolarized [U-^2^H, 2-^13^C]­fructose and *in vivo* dynamic slice-localized spectroscopy was performed. The single repetition spectra of hyperpolarized spectroscopic data demonstrated elevated F1P labeling in the fed state with reduced labeling in the fasted state (Figure 2C). This qualitative observation was quantified and normalized to the total precursor and F1P signal and demonstrated greater F1P accumulation in the fed state (Figure 2D). This indicates two possibilities, one is that the fed state potentially upregulates KHK activity and fructose metabolism, or two, the fasted state oxidizes fructose carbons to maintain the energetic demands of glucagon induced gluconeogenesis.

**Figure 2:**
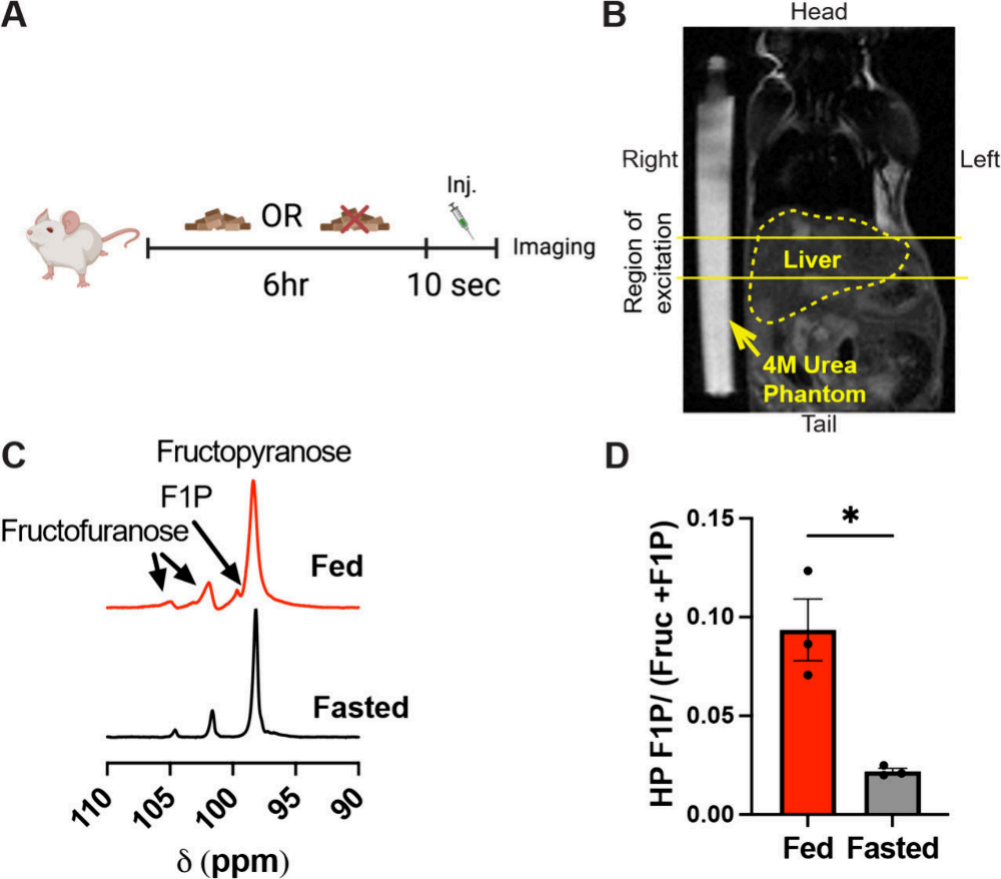
*In vivo* magnetic resonance spectroscopy demonstrates increased fructose-1-phosphate accumulation in the fed mouse liver. (A) Experimental timeline involved *ad libitum* access to food or fasting for 6 h prior to ^1^H magnetic resonance imaging (MRI) and the injection of hyperpolarized [U-^2^H, 2-^13^C]­fructose followed by dynamic ^13^C spectroscopy. (B) Anatomical ^1^H MRI coronal *T*_2_ weighted spin echo images. Outlined in a red dashed line is the contour of the liver and outlined in solid red lines is the region of slice excitation. A 4 M ^13^C urea phantom was placed on the right side of the animals. (C) Representative single repetition spectra acquired after the injection of hyperpolarized [U-^2^H, 2-^13^C]­fructose demonstrating the detection of furanose and pyranose isoforms as well as the production of [U-^2^H, 2-^13^C]­F1P. (D) Summed spectral analysis in which greater ratios of F1P to the sum of fructose and F1P were detected in the fed state indicating greater accumulation of F1P compared to the fasted state.

## NMR Reveals Increased Lactate and Alanine Production from [2-^13^C]­Fructose in Fasted Livers

To assess the fate of fructose carbons in fed and fasted states, a parallel cohort of mice were injected with [2-^13^C]­fructose followed by the extraction of the liver 1 min postinjection and analyzed by NMR. Qualitative spectral analysis and quantification of fructopyranose and F1P confirm elevated F1P pool sizes in the fed state (Figure 3A,B). In contrast to F1P pool sizes, increased labeling in the C_2_ position of lactate and alanine was detected in the fasted state (Figure 3C,D), thus suggesting that the fasted liver utilizes fructose carbons for downstream metabolic processes that may potentially sustain the metabolic demands of gluconeogenesis triggered by a fasting glucagon response. This effect is insensitive to pool size changes as no differences in lactate and alanine pools were detected between fed and fasted states (Figure S9).

**Figure 3:**
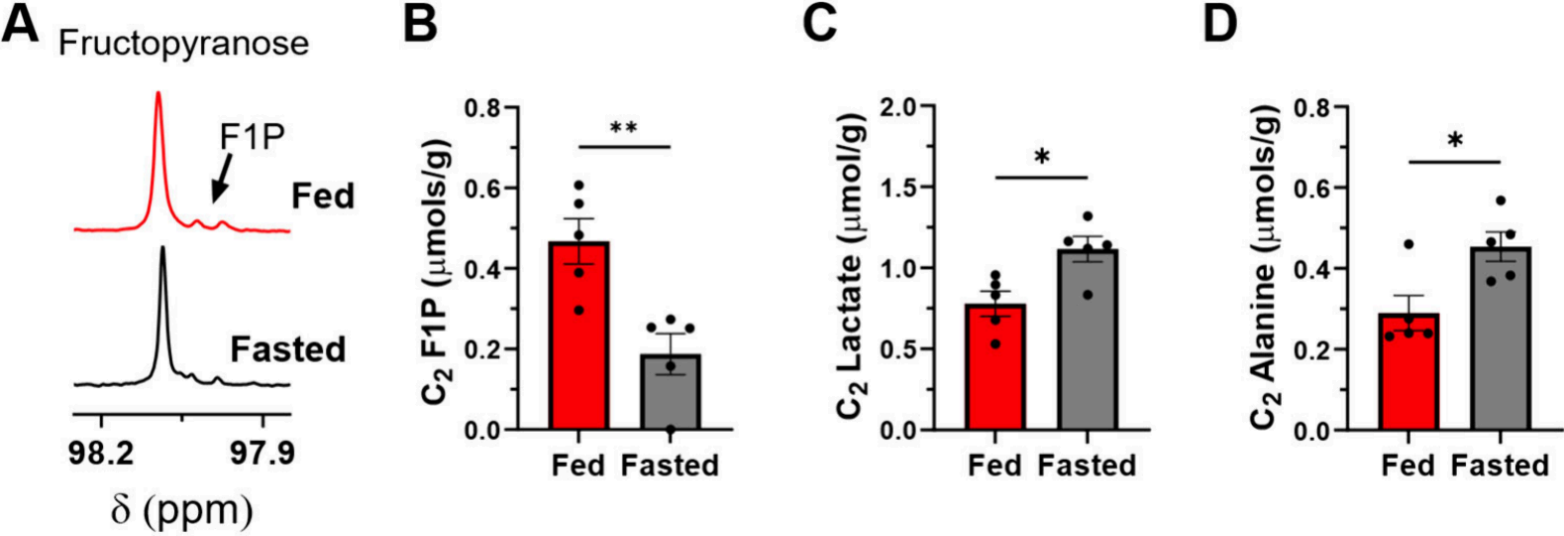
Increased fructose utilization under fasted conditions detected by nuclear magnetic resonance spectroscopy of liver tissue. (A) *Ex vivo* analysis of ^13^C labeling by nuclear magnetic resonance (NMR) detected elevated F1P labeling in the fed state. Quantification of spectroscopic data demonstrated significantly reduced F1P labeling (B) and significantly increased lactate (C) and alanine (D) labeling in the fasted state.

## Fructose Metabolism Is Dominated by Gluconeogenesis in Fed Livers

To gain further insights into the specific metabolic pathways that fructose carbons enter, we performed another set of animal experiments that traced the utilization of [U-^13^C]­fructose (Figure 4A) in fed and fasted states with LC–MS. Hierarchical heat map clustering analysis of fractional enrichments demonstrates distinct metabolic profiles between states (Figure 4B). More specifically, we observe enhanced labeling in TCA cycle intermediates like *m* + 2 and *m* + 3 citrate and α-ketoglutarate in the case of fasting, indicating active flux through both pyruvate dehydrogenase and pyruvate carboxylase reactions (Figure 4 and Figure S10). In combination with elevated pyruvate labeling under fasting conditions (Figure 4C), these data clearly indicate that fructose tracing is sensitive to a fasting induced a dominance toward downstream metabolism. Additionally, we also detected enhanced gluconeogenesis in the fed state as evidenced by significantly greater *m* + 3 glucose in plasma compared to fasting conditions (Figure 4E). This suggests that in post prandial states, fructose can replenish gluconeogenic precursors even when insulin stimulates glucose oxidation. Elevated *m* + 3 lactate in plasma is also observed in the fed state (Figure 4F).

**Figure 4:**
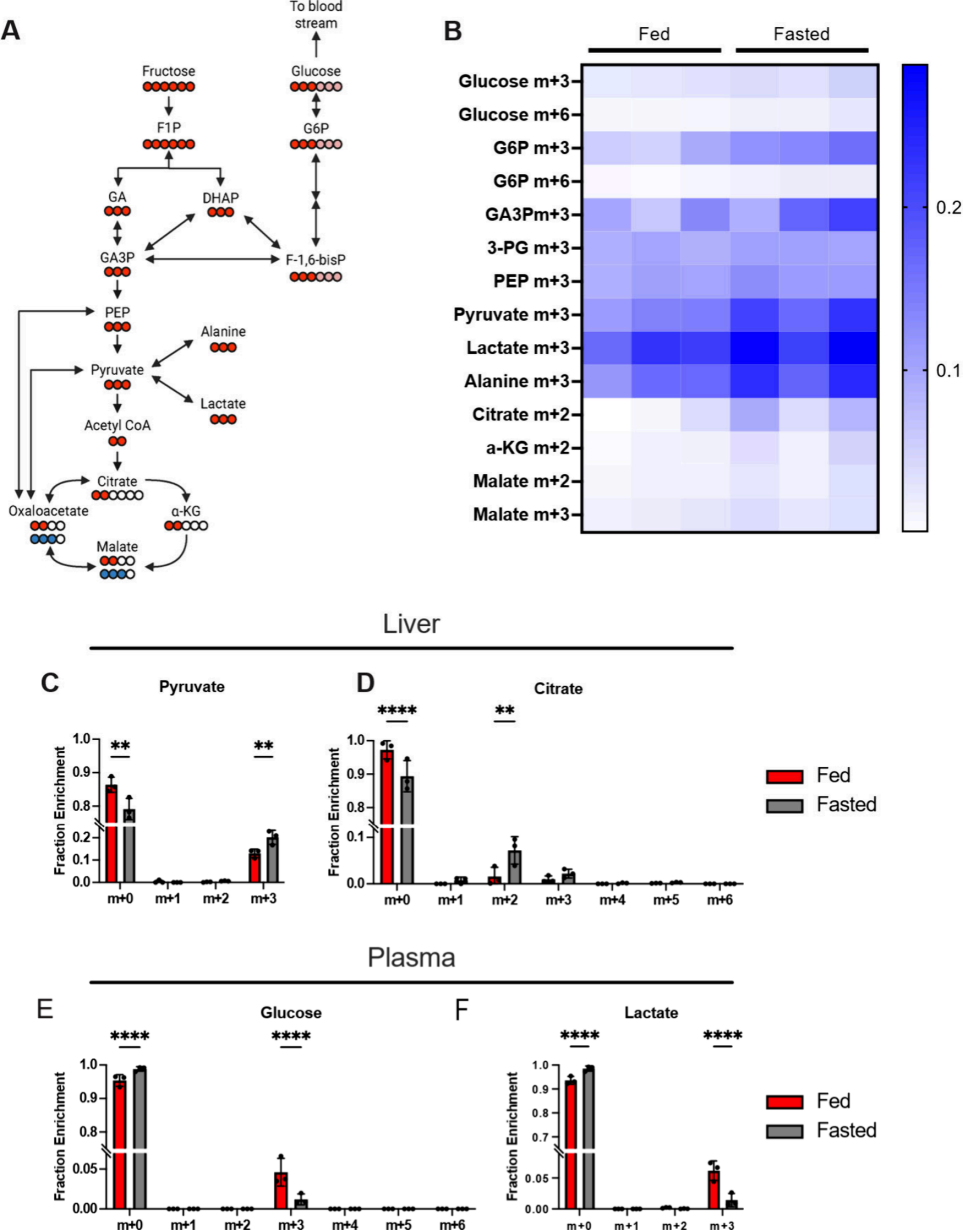
*In vivo* [U-^13^C]­fructose tracing reveals hepatic gluconeogenesis in the fed state and increased downstream metabolism in the fasted state. (A) Labeling diagram highlighting the deposition of ^13^C label from fructose to downstream glycolytic and TCA cycle intermediates, deposition into gluconeogenic metabolites, as well as contribution from pyruvate carboxylase flux (blue circles). (B) Heat map analysis of primary isotope mass species generated from [U-^13^C]­fructose indicates greater TCA cycle activity in the fasted state. Mass isotopologue distributions of hepatic pyruvate (C) and citrate (D), as well as plasma glucose (E) and lactate (F) demonstrate gluconeogenic synthesis of fructose derived glucose in the fed state, as well as enhanced downstream metabolism in the fasted state evident by greater labeling on pyruvate and citrate.

Overall, we demonstrate the ability of hyperpolarized [U-^2^H, 2-^13^C]­fructose to discriminate F1P production in fed and fasted states, respectively, with subsequent nonpolarized ^13^C labeled fructose experiments demonstrating a gluconeogenic dominance over downstream metabolism in the fed state. This is a new application of this probe and establishes the foundation of a paradigm potentially capable of noninvasively assessing and monitoring metabolic disease in the liver that will need to overcome limitations in *T*_1_ and polarization to generate clinically useful images of fructose utilization. Currently, 3T clinical hyperpolarized imaging with ^13^C pyruvate can achieve *T*_1_ values greater than 90 s and polarization percentages greater than 35%, thus setting important target benchmarks for newer probes like fructose. While advancements will certainly be required to increase the *T*_1_ and polarization of hyperpolarized fructose, we believe that further technological development in cryogenic probes and pulse sequences will help attain clinically useful data and images with *T*_1_ values and polarization lower than those of ^13^C pyruvate. Additionally, new studies must consider the assessment of both fed and fasted states, especially in the context of fructolytic gluconeogenesis as it was only detectable in the fed state. This is an important consideration for clinical translation as majority of laboratory testing and imaging examinations occur during the fasted state. Furthermore, this study prompts further testing of hyperpolarized [U-^2^H, 2-^13^C]­fructose as a probe to image the transition of healthy liver to MASLD, metabolic dysfunction-associated steatohepatitis (MASH), and eventually hepatocellular carcinoma (HCC). A specific, noninvasive imaging method for liver disease diagnosis and staging would be of great benefit as ∼30% of the US population is currently affected by liver related morbidities.^35^ Future developments of this technology may be essential for improving our ability to image disease and improve patient health care outcomes through accurate detection and monitoring.

## Methods

### Chemical Synthesis of [U-^2^H, 2-^13^C]­Fructose

Synthesis of [U-^2^H, 2-^13^C]­fructose was achieved through protection, deuteration, and deprotection reactions. Protection was performed by first dissolving 2.0 g of [2-^13^C]-D-fructose (Figure 1, **1a** and **1b**) in 100 mL of HPLC grade methanol using a 250 mL Erlenmeyer flask. Afterward, 100 μL of acetyl chloride (110.4 mg, 1.406 mmol, 0.15 equiv) was added, the reaction flask was sealed, and the mixture was stirred at RT (RT) overnight. Next, equal amounts of Amberlite IRA-410 OH resin were added and the mixture was stirred for an additional 15 min. After stirring, the reaction mixture was collected and filtered, and then the remaining resin was thoroughly washed with HPLC grade methanol. The solvent was retained and evaporated to yield a **2** as a clear oil (Figure 1). The resulting oil was then directly used as a starting compound in the deuteration reaction which was adopted from previously published methodology.^36^ In brief, 1.04 g (5.33 mmol) of **1** (Figure 1) was combined with 21.3 mL of 99.9% D_2_O in a 100 mL round-bottom flask. Then 2.1 g of 5 wt % of Ru/C was added to the reaction mixture followed by the addition of 85 mg (2.13 mmol, 0.4 equiv) of freshly ground NaOH. The reaction mixture was then flash frozen in liquid N_2_, thawed, and bubbled with H_2_ for 15 min under vacuum to remove excess oxygen from the reaction mixture. This was followed by constant stirring in the presence of H_2_ at 80 °C for 2–3 4 h cycles. Afterward, the complete reaction mixture was filtered and rinsed with H_2_O (100 mL). DOWEX 50WX8 hydrogen form resin was then added to adjust pH to 7. The final solution was collected by filtering out the resin, followed by lyophilization. Deuteration was repeated at least two times or until deuterium deposition in **3** (Figure 1) was measured to be ≥96% by NMR. Next, 367 mg (1.96 mmol) of **3** (Figure 1) were combined with 3.9 mL of 1 mM HCl in H_2_O. Using a water-cooled Dean–Stark trap, the reaction mixture was refluxed for 3 h. Then the reaction was chilled and pH adjusted to 7 with IRA-410 OH resin. The final mixture was then filtered and dried with a lyophilizer to generate a clear foam.

### Dissolution Dynamic Nuclear Polarization

A 5.0 T SpinLab Hyperpolarizer (GE Healthcare) was utilized to perform dissolution dynamic nuclear polarization (dDNP). A sample of 5 M deuterated [2-^13^C]­fructose (Figure 1, **4a** and **4b**) in water with 15 mM OX063 trityl radical (Oxford Instruments) was polarized for 2 h. The sample was then released from the polarizer by instantaneous solvation with an excess of D_2_O heated to 130 °C, generating a final solution of ∼50 mM deuterated [2-^13^C]­fructose at a neutral pH.

### *T*_1_ Measurements of Hyperpolarized [2-^13^C]­Fructose

Analysis and quantification of polarization was performed immediately following sample dissolution. One mL of hyperpolarized sample was rapidly transferred to a 5 mm NMR tube and inserted into a 1 T Spinsolve 13C NMR spectrometer (Magitrek, NZ). Spectral acquisition was achieved with 5° excitation pulses applied every 3 s for over 3 min. Dynamic data was processed using Mnova (Mestrelab, ES) by integrating across the spectral boundaries of each hyperpolarized peak. Integrals were utilized to estimate apparent relaxation time (*T*_1_) by fitting a monoexponential curve that was corrected for flip angle and timed delays between the start of dissolution to the start of spectral acquisition. Thermal polarization was acquired with 90° pulses every 10 s for a total of 1024 averages. After corrections, the final polarization values were estimated to be 10–15% for all dissolutions. To quantify substrate concentrations, samples were spiked with 1 mM Gd-DOTA and a 100 mM [1-^13^C] lactate internal standard followed by ^13^C NMR analysis at 14.1 T. Integrals of the C_2_ carbon of fructose and lactate standard were compared to estimated concentrations.

### *In Vivo* Hyperpolarized Magnetic Resonance Spectroscopy

All animal experiments performed were reviewed and approved by the Institutional Animal Care and Use Committee at Memorial Sloan Kettering Cancer Center under protocol number 13-12-019. First, mice (8–9 weeks old) were subjected to a 6 h period of *ad libitum* access to food or fasting. After this period, mice were anesthetized with 1.5% and tail veins catharized with a catheter (Braintree Scientific, USA) preloaded with heparinized (10 U/ml) saline solution used to avoid potential coagulation and blockage of the catheter line. After catheterization, the mice were positioned within a 3 T magnetic resonance imaging (MRI) scanner (Bruker, US) equipped with a dual tune ^1^H/^13^C volume coil. A 4 M [^13^C]­urea phantom was secured on the right side of each mouse, and each animal was positioned to center its liver within the center of the coil and scanner. For anatomical referencing, coronal ^1^H *T*_2_ weighted spin echo images were acquired prior to slice selective spectroscopy. Based on the ^1^H images, a selective slice (5 mm) was placed in the central region of the liver. After slice localization was completed, the hyperpolarized [2-^13^C]­fructose sample was ejected from the polarizer by dissolution and 20 s later was injected (350 μL) into the tail vein of each animal over a period of 10 s. Dynamic spectral data was acquired with an excitation flip angle of 30°, spectral width of 2,564 Hz, and 2048 points were used every 1 s over a 2 min period starting at the same time as dissolution.

### *In Vivo* [2-^13^C]­Fructose Tracing and Nuclear Magnetic Resonance Analysis

In a separate cohort of animals, [2-^13^C]­fructose (4 g/kg of body weight) was administered via intraperitoneal injection in benchtop experiments, mimicking the exact same conditions of the hyperpolarized experiments. After 1 min post tracer administration, animals were euthanized for blood and liver tissue collection for subsequent analysis. Approximately 200 mg of liver was dissected from at least three different regions of the organ. The tissue was then added to a bead mill tube with perchloric acid (4%; 1:4, w/v) and finely ground using the Bead Ruptor 24 Bead Mill Homogenizer (Omni International Inc.) for 3 min. Then, tubes were centrifuged, and the supernatant containing metabolites was collected and mixed with chloroform/tri-*n*-octylamine (78%/22%, v/v) and centrifuged a second time. The aqueous layer was collected, frozen, and lyophilized prior to NMR analysis. For NMR analysis, samples were resuspended in 600 μL of D_2_O containing 10 mM imidazole, 0.2 wt % sodium azide, and 1 mM sodium trimethylsilylpropanesulfonate (DSS) as a concentration reference standard. Samples had slightly basic pH (∼7.9), causing the F1P peaks to shift upfield and appear to the right of the fructopyranose peak. Carbon and proton NMR spectra were acquired on a 14,1-T NMR spectrometer (Bruker Biospin), and data was processed using Mnova (Mestrelab, ES).

### *In Vivo* [U-^13^C]­Fructose Tracing and Mass Spectrometry Analysis

Under the same conditions as previously described, one last cohort of mice was injected intraperitoneally with [U-^13^C]­fructose (4 g/kg of body weight), followed by euthanasia and collection of liver and plasma 1 min postinjection. Tissue and plasma were snap-frozen before further analysis. Targeted LC–MS analysis was performed exactly as in previously described methods.

### Statistical Analysis

Column plots were statistically assessed with a two-tailed, unpaired Student’s *t* test. Grouped plots were analyzed using a two-way analysis of variance (ANOVA) with the idák multiple-comparison correction. *P* values were calculated and comparisons with *P* < 0.05 were considered to be statistically significant. Hierarchical heat map clustering was performed with GraphPad prism.

## Supplementary Material



## Notes

^1^H and ^13^C NMR spectra for all compounds, pool sizes of lactate and alanine measured by NMR, and mass isotopologue distributions (MIDs) of all measured metabolites by LC-MS (PDF)
