# Supplementary material for: Hyperpolarized [U‑2H, 2-13C]Fructose Distinguishes Direct Hepatic Gluconeogenesis Through Fructose-1-Phosphate Production in Fed and Fasted States
Source: ACS Chem Biol. 2026 Mar 4;21(3):423–30. doi: 10.1021/acschembio.5c00980 (PMC13010333; doi:10.1021/acschembio.5c00980)
Supplement: Supplementary file 1 [file cb5c00980_si_001.pdf]

Supporting Information

Hyperpolarized [U-^{2}H, 2-^{13}C]Fructose Distinguishes Hepatic Gluconeogenesis and Oxidative Metabolism in Fed and Fasted States

Celia Martínez de la Torre[1], Grace Figlioli[1], Mario C. Chang[1], Quinlan Cullen[1,2,3], and Kayvan R. Keshari[1,2,3,*].

[1]Department of Radiology, Memorial Sloan Kettering Cancer Center, New York, NY, USA.

[2]Molecular Pharmacology Program, Memorial Sloan Kettering Cancer Center, New York, NY, USA.

[3]Weill Cornell Medical College, New York, NY, USA.

***Corresponding Author:** Kayvan R. Keshari, rahimikk@mskcc.org

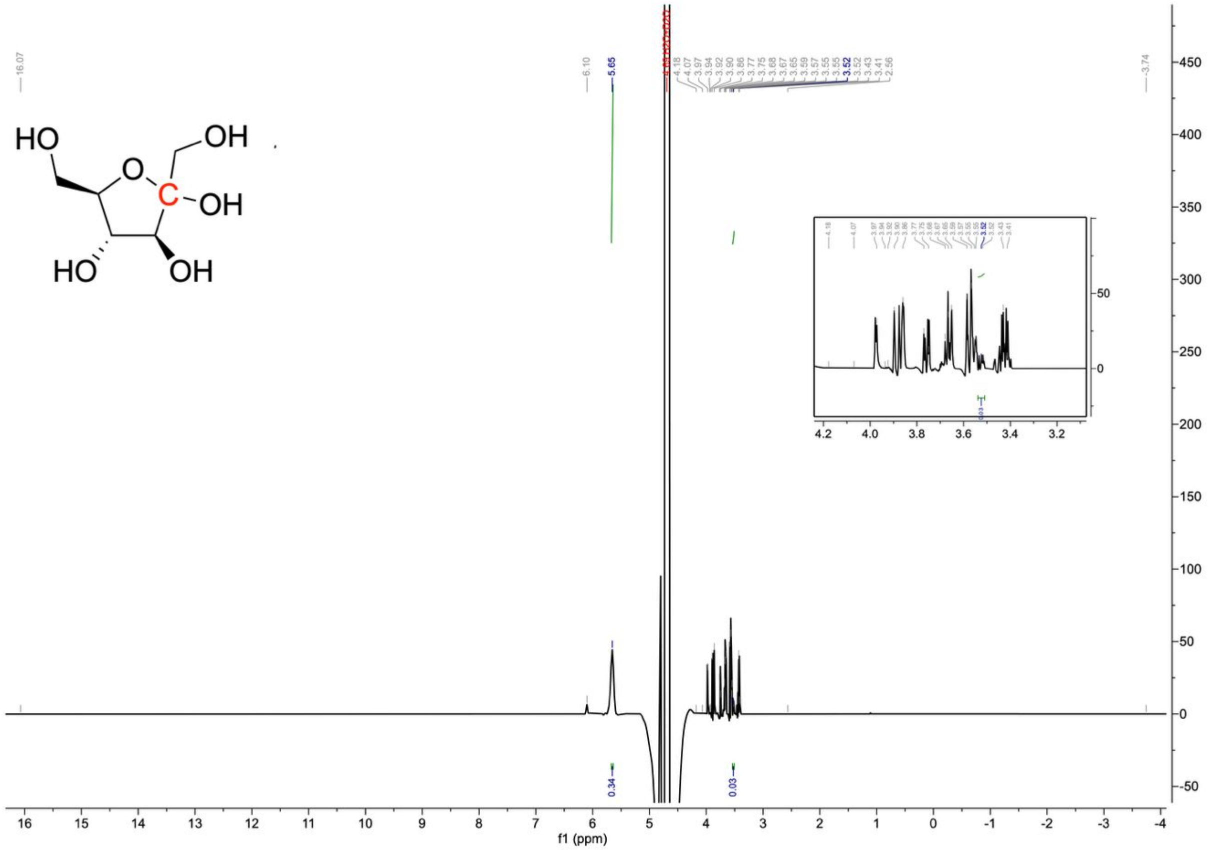

Figure S1. 14.1T ^{1}H-NMR of spectra Compound 1, [2-^{13}C]-fructose. ^{1}H NMR (600 MHz, H_2O) δ(ppm): 16.070, 6.100, 5.653, 4.693, 4.178, 4.069, 3.972, 3.936, 3.923, 3.897, 3.859, 3.770, 3.750, 3.679, 3.666, 3.651, 3.586, 3.567, 3.553, 3.548, 3.524, 3.519, 3.430, 3.413, 2.563, -3.745.

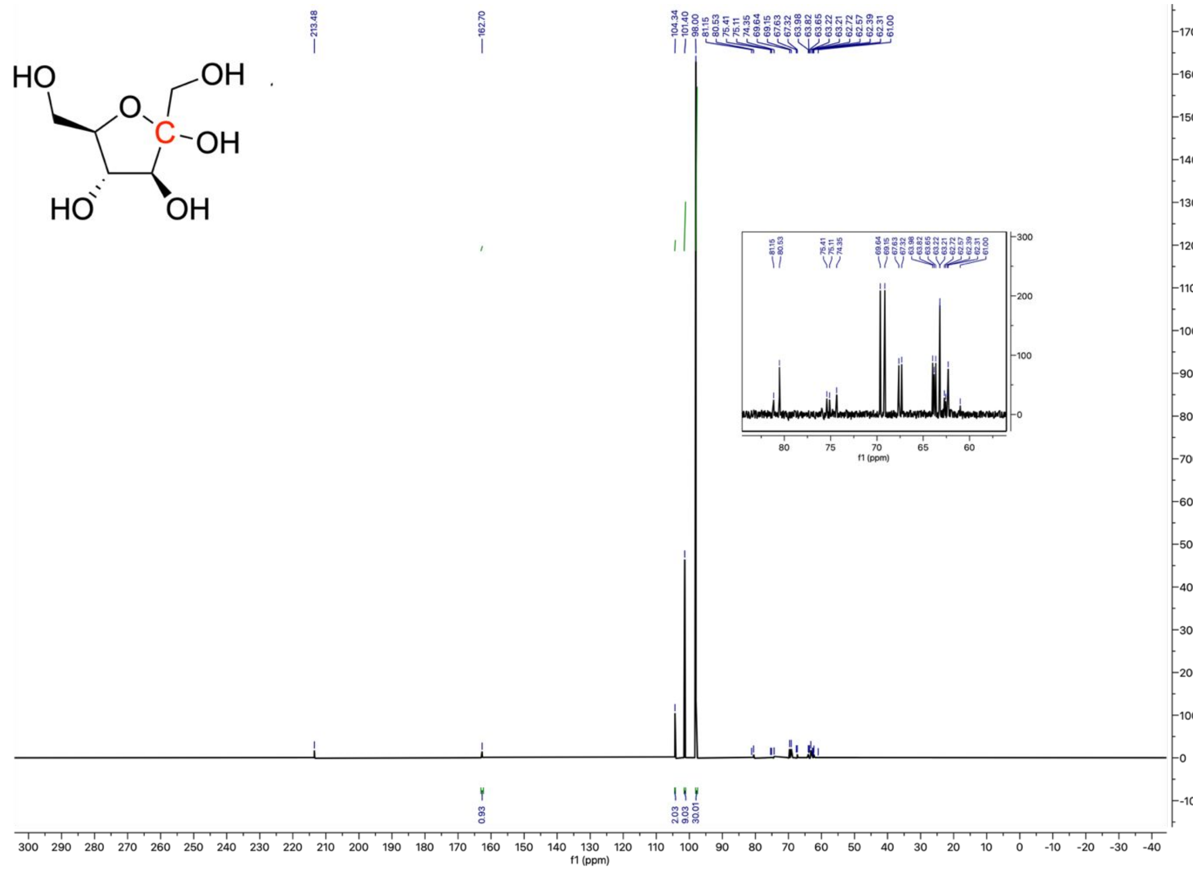

Figure S2. 14.1T ^{13}C-NMR spectra of Compound 1, [2-^{13}C]-fructose. ^{13}C NMR (600 MHz, H_2O) δ (ppm): 213.478, 162.703, 104.341, 101.398, 98.000, 81.151, 80.529, 75.410, 75.107, 74.354, 69.639, 69.148, 67.634, 67.320, 63.978, 63.821, 63.648, 63.221, 63.207, 62.725, 62.566, 62.386, 62.311, 61.000.

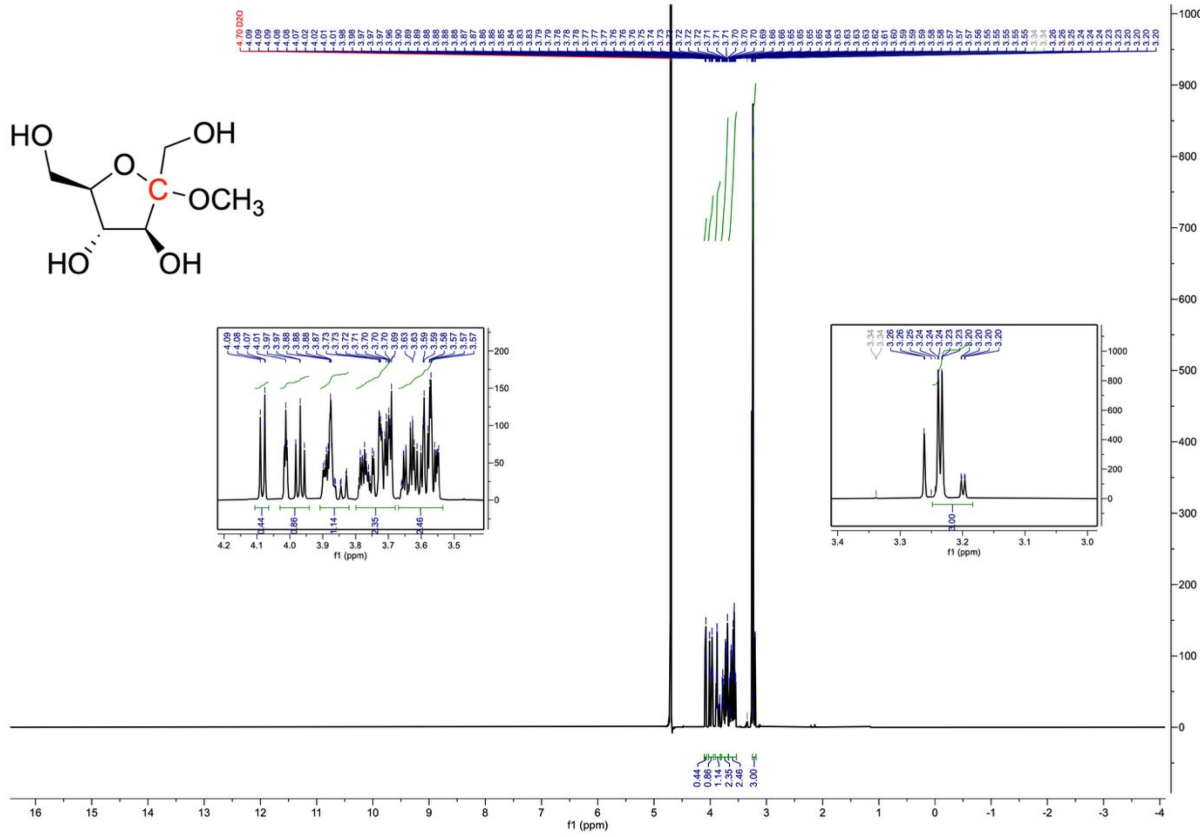

Figure S3. 14.1T ^{1}H-NMR spectra of Compound 2, [2-^{13}C]-methyl-D-fructofuranoside. ^{1}H NMR (600 MHz, D_2O) δ (ppm): 4.701, 4.091, 4.090, 4.089, 4.077, 4.076, 4.075, 4.017, 4.016, 4.012, 4.008, 3.982, 3.981, 3.970, 3.969, 3.968, 3.955, 3.899, 3.894, 3.889, 3.884, 3.880, 3.879, 3.876, 3.874, 3.871, 3.864, 3.861, 3.846, 3.844, 3.829, 3.827, 3.790, 3.785, 3.780, 3.779, 3.777, 3.773, 3.768, 3.766, 3.764, 3.760, 3.755, 3.749, 3.744, 3.729, 3.727, 3.724, 3.721, 3.719, 3.712, 3.710, 3.706, 3.701, 3.699, 3.695, 3.691, 3.661, 3.658, 3.654, 3.653, 3.647, 3.646, 3.640, 3.634, 3.633, 3.627, 3.626, 3.621, 3.613, 3.601, 3.595, 3.594, 3.591, 3.580, 3.575, 3.574, 3.571, 3.568, 3.559, 3.555, 3.554, 3.551, 3.550, 3.548, 3.339, 3.338, 3.262, 3.261, 3.250, 3.244, 3.239, 3.238, 3.233, 3.232, 3.203, 3.201, 3.197, 3.196.

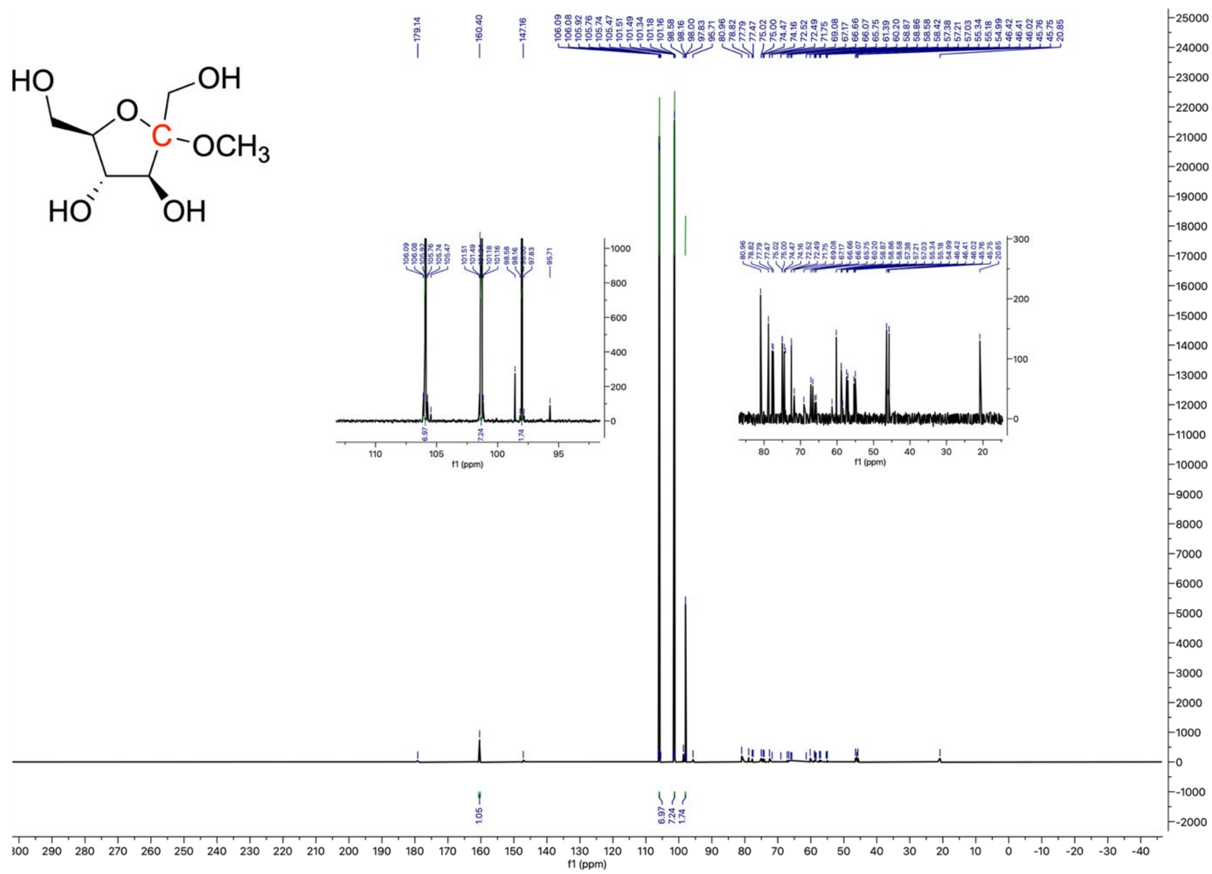

Figure S4. 14.1T ^{13}C-NMR spectra of Compound 2, [2-^{13}C]-methyl-D-fructofuranoside. ^{13}C NMR (600 MHz, D_2O) δ (ppm): 179.143, 160.401, 147.160, 106.093, 106.077, 105.925, 105.756, 105.742, 105.467, 101.512, 101.493, 101.345, 101.182, 101.164, 98.577, 98.161, 98.001, 97.827, 95.707, 80.965, 78.818, 77.789, 77.468, 75.022, 75.003, 74.467, 74.155, 72.517, 72.491, 71.746, 69.079, 67.172, 66.663, 66.073, 65.752, 61.392, 60.198, 58.872, 58.856, 58.583, 58.421, 57.377, 57.213, 57.030, 55.340, 55.176, 54.989, 46.420, 46.405, 46.019, 45.761, 45.747, 20.848.

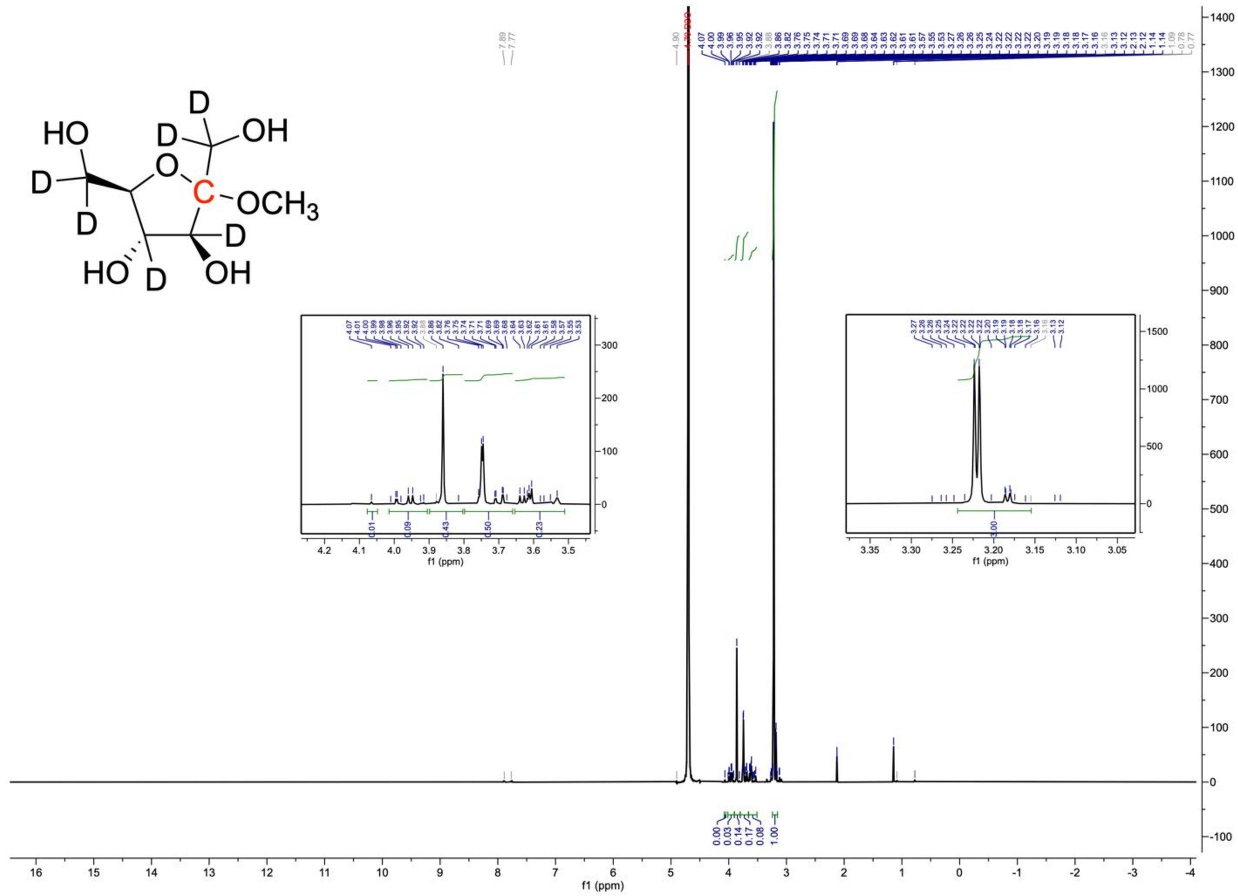

Figure S5. 14.1T ^{1}H-NMR spectra of Compound 3, [1,1,3,4,6,6-$^2H_6$, 2-^{13}C]-methyl-D-furanoside. ^{1}H NMR (600 MHz, D_2O) δ(ppm): 7.886, 7.766, 4.902, 4.701, 4.065, 4.010, 3.995, 3.992, 3.981, 3.960, 3.947, 3.924, 3.916, 3.879, 3.860, 3.816, 3.758, 3.749, 3.745, 3.711, 3.708, 3.690, 3.687, 3.677, 3.639, 3.627, 3.618, 3.613, 3.606, 3.581, 3.570, 3.552, 3.532, 3.275, 3.264, 3.258, 3.248, 3.235, 3.224, 3.223, 3.218, 3.217, 3.203, 3.186, 3.185, 3.180, 3.179, 3.175, 3.161, 3.155, 3.126, 3.119, 2.125, 2.124, 1.144, 1.143, 1.085, 0.781, 0.770.

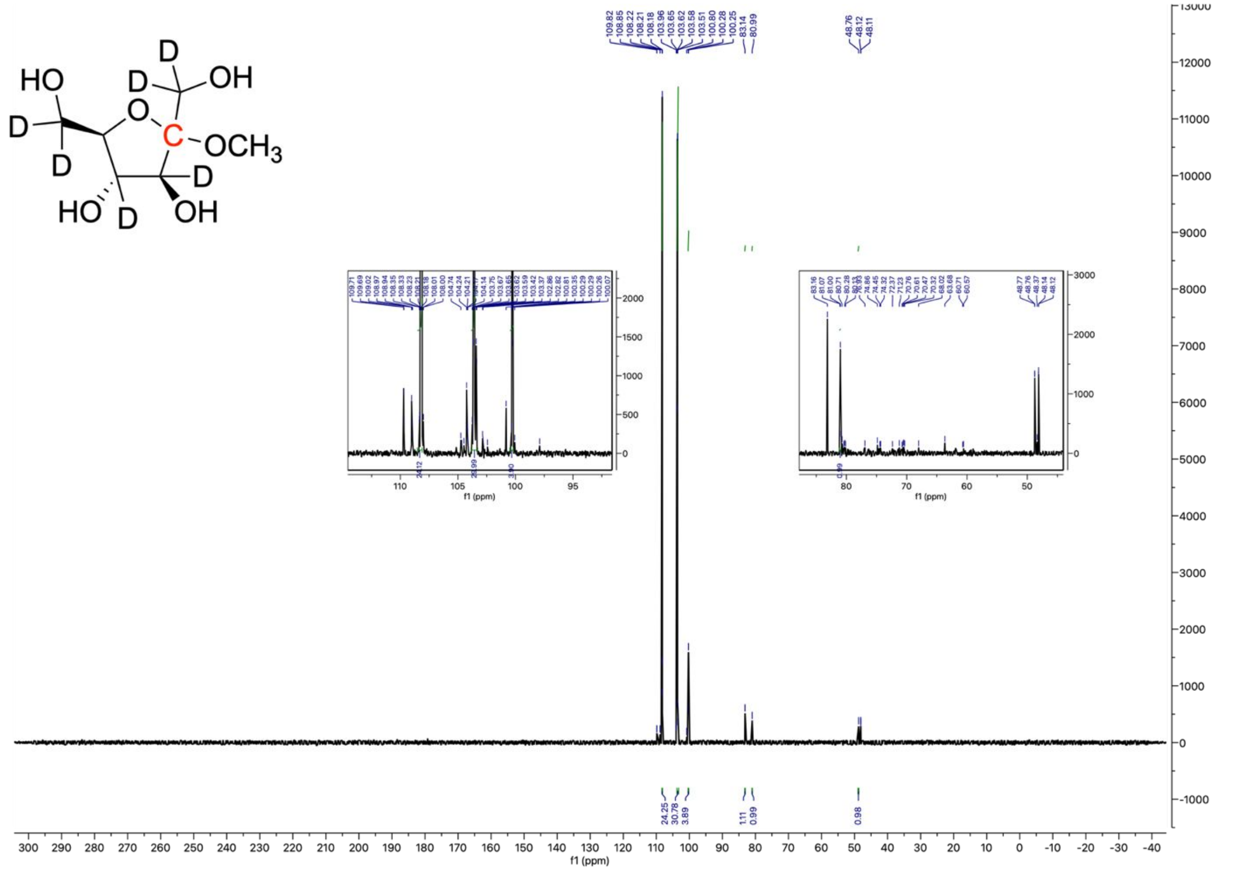

Figure S6. 14.1T ^{13}C NMR spectra of Compound 3, [1,1,3,4,6,6-$^{2}H_6$, 2-^{13}C]-methyl-D-furanoside. ^{13}C NMR (600 MHz, D_2O) δ (ppm): 178.052, 177.968, 174.104, 109.714, 109.686, 109.022, 108.973, 108.943, 108.345, 108.330, 108.226, 108.214, 108.177, 108.010, 107.995, 104.741, 104.664, 104.477, 104.243, 104.209, 104.172, 104.141, 103.754, 103.666, 103.654, 103.622, 103.587, 103.415, 103.370, 102.860, 102.822, 102.437, 100.809, 100.355, 100.294, 100.285, 100.257, 100.069, 97.904, 83.155, 81.073, 81.002, 80.714, 80.426, 80.277, 80.130, 76.927, 74.860, 74.449, 74.315, 72.370, 71.231, 70.758, 70.614, 70.473, 70.324, 68.016, 63.678, 60.713, 60.566, 48.773, 48.758, 48.371, 48.138, 48.123.

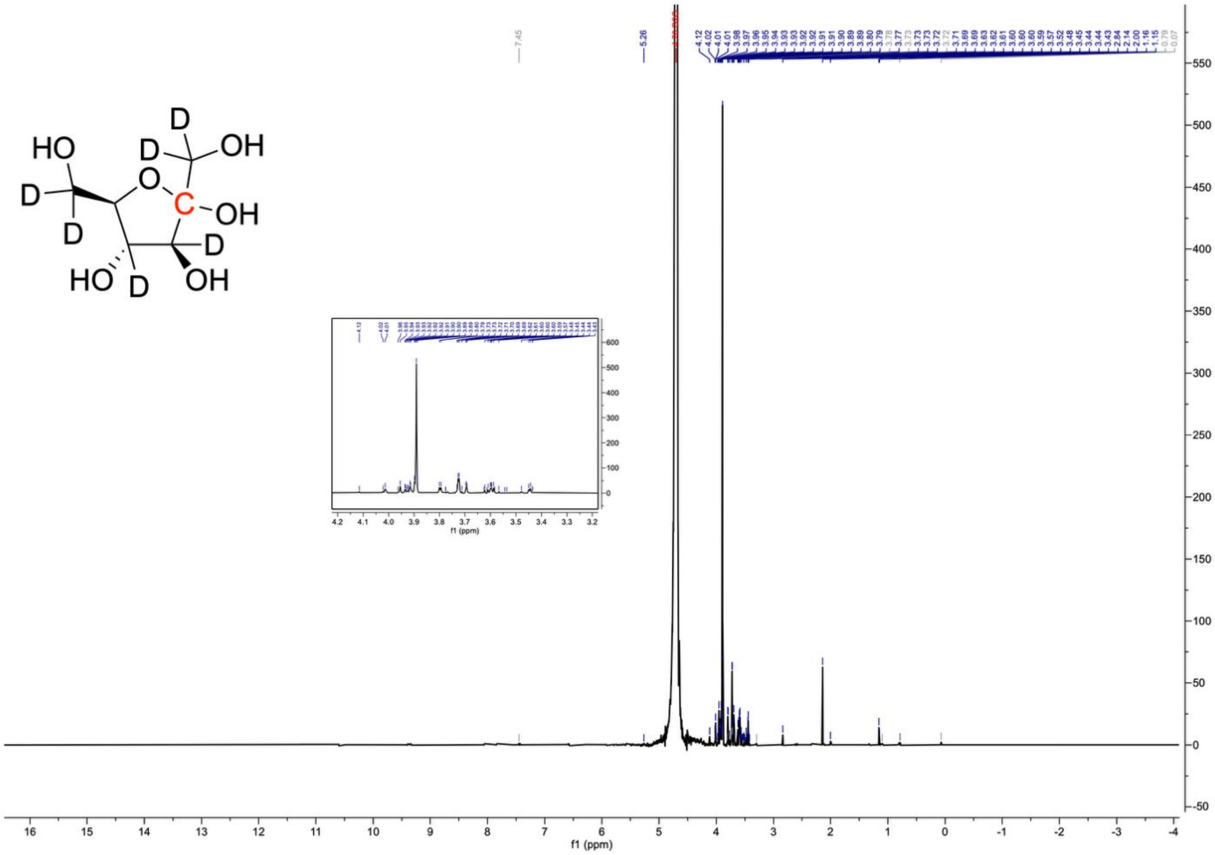

Figure S7. 14.1T ^{1}H-NMR spectra of Compound 4, [1,1,3,4,6,6-$^{2}H_{6}$, 2-^{13}C]-D-fructose. ^{1}H NMR (600 MHz, D_2O) δ (ppm): 7.449, 5.263, 4.701, 4.115, 4.022, 4.014, 4.010, 3.976, 3.974, 3.963, 3.955, 3.937, 3.934, 3.930, 3.925, 3.916, 3.913, 3.908, 3.898, 3.894, 3.891, 3.811, 3.800, 3.794, 3.776, 3.768, 3.744, 3.734, 3.731, 3.726, 3.722, 3.718, 3.711, 3.695, 3.691, 3.671, 3.625, 3.622, 3.609, 3.600, 3.597, 3.595, 3.587, 3.566, 3.557, 3.544, 3.536, 3.521, 3.516, 3.504, 3.478, 3.472, 3.450, 3.443, 3.436, 3.435, 3.421, 3.298, 2.836, 2.138, 2.001, 1.156, 1.146, 1.098, 0.793, 0.782, 0.069.

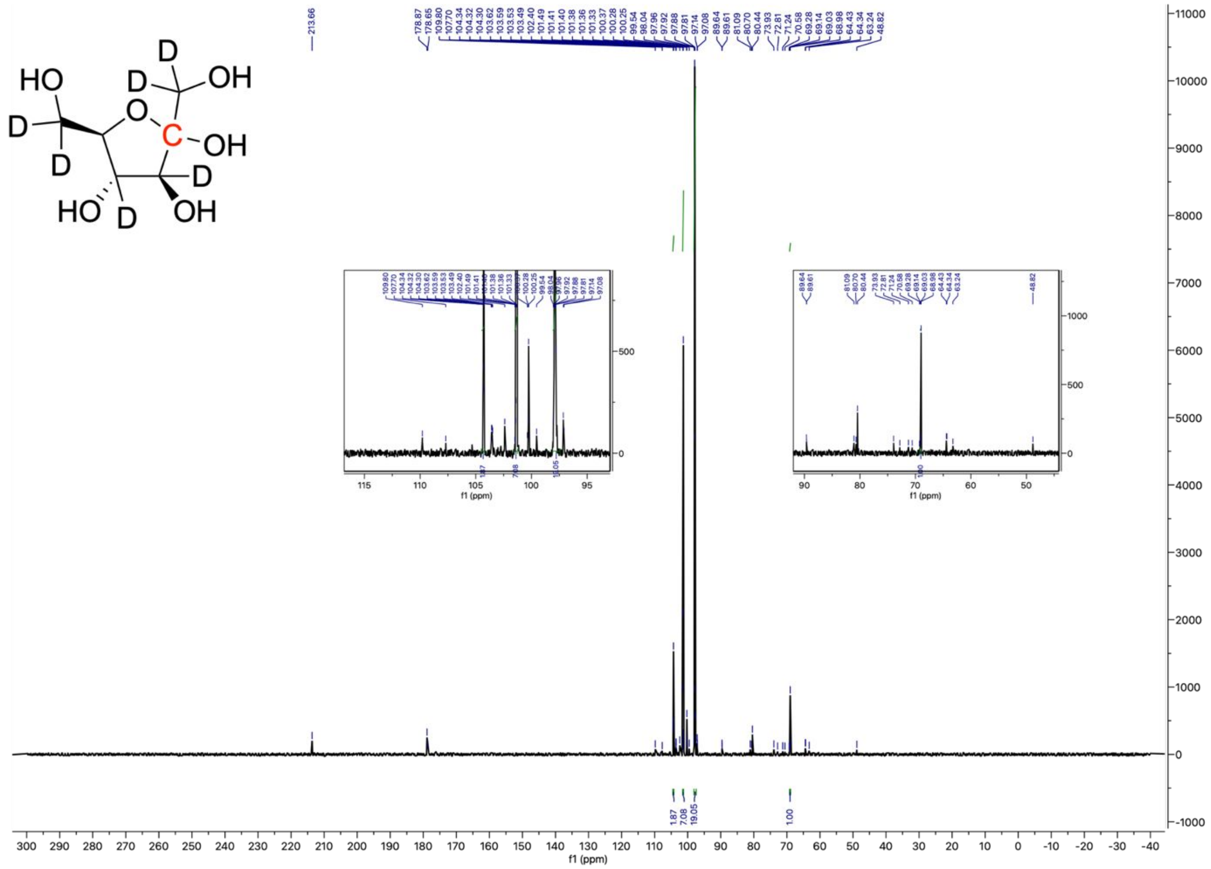

Figure S8. 14.1T ^{13}C-NMR spectra of Compound 4, [1,1,3,4,6,6-$^{2}H_6$, 2-^{13}C]-D-fructose. ^{13}C NMR (600 MHz, D_2O) δ (ppm): 213.656, 178.871, 178.647, 109.798, 107.697, 104.337, 104.318, 104.296, 103.621, 103.585, 103.530, 103.485, 102.404, 101.490, 101.411, 101.397, 101.376, 101.364, 101.328, 100.365, 100.282, 100.254, 99.541, 98.039, 97.957, 97.923, 97.884, 97.808, 97.140, 97.081, 89.645, 89.609, 81.089, 80.704, 80.439, 73.933, 72.813, 71.243, 70.578, 69.283, 69.139, 69.026, 68.979, 64.425, 64.337, 63.238, 48.822.

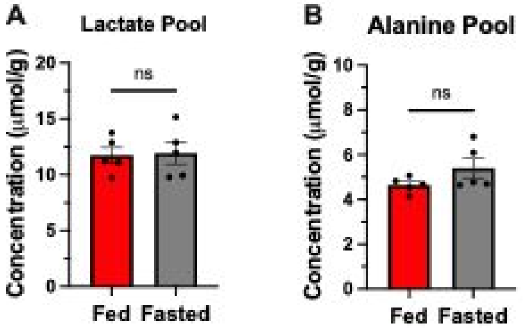

Figure S9. Pool sizes show no difference between fed and fasted states. *Ex vivo* NMR analysis of ^{13}C labeling detected no difference in (**A**) lactate and (**B**) alanine pool sizes across different prandial states.

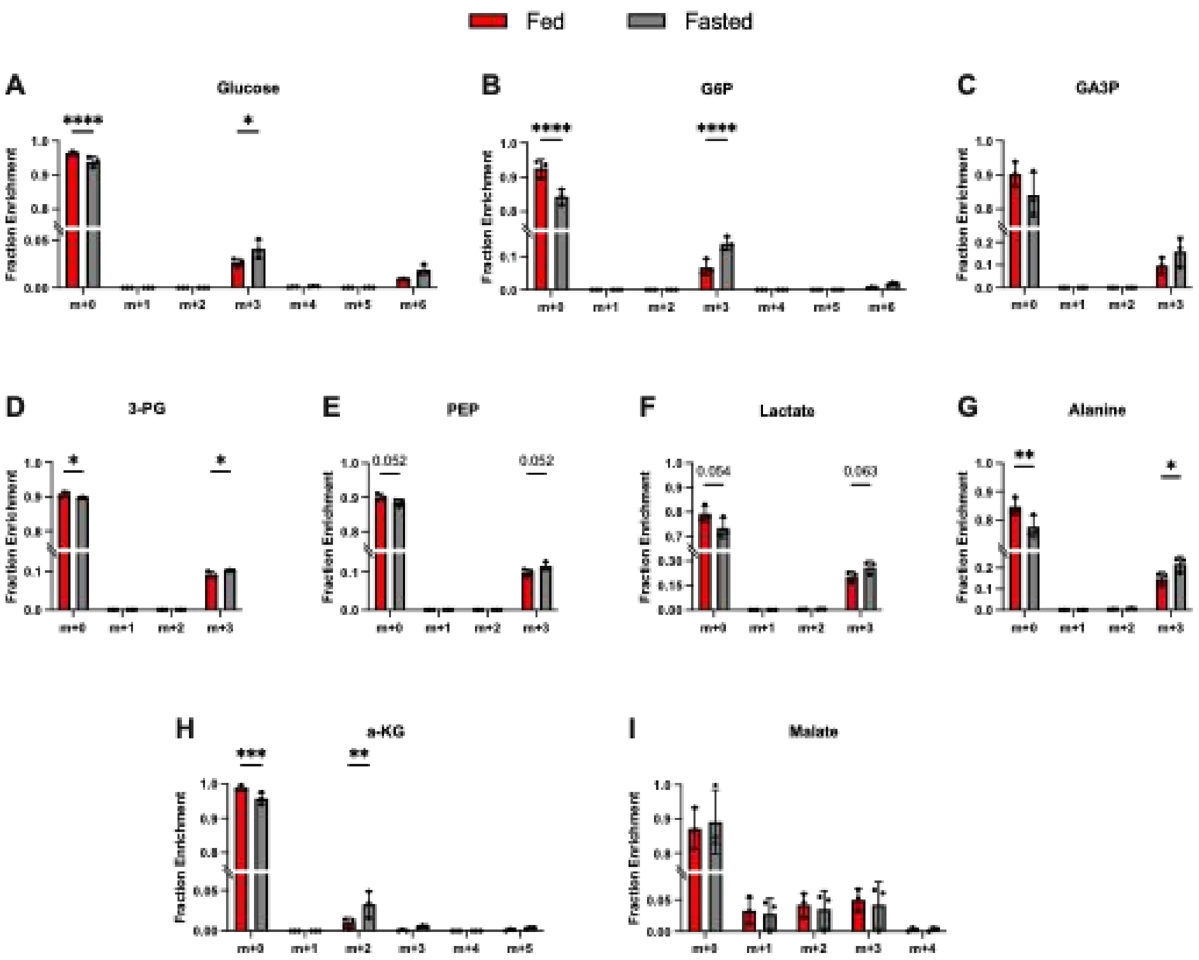

Figure S10. Fasting causes greater TCA cycle activity in the liver. Mass isotopologue distribution of hepatic metabolites from [U-^{13}C]fructose tracing.
